# Functional and neuromuscular changes induced *via* a low-cost, muscle-computer interface for telerehabilitation: A feasibility study in chronic stroke

**DOI:** 10.3389/fnrgo.2022.1046695

**Published:** 2022-11-17

**Authors:** Octavio Marin-Pardo, Miranda Rennie Donnelly, Coralie S. Phanord, Kira Wong, Jessica Pan, Sook-Lei Liew

**Affiliations:** ^1^Department of Biomedical Engineering, University of Southern California, Los Angeles, CA, United States; ^2^Chan Division of Occupational Science and Occupational Therapy, University of Southern California, Los Angeles, CA, United States; ^3^Dornsife College of Letters, Arts, and Sciences, University of Southern California, Los Angeles, CA, United States; ^4^Stevens Neuroinformatics Institute, Department of Neurology, University of Southern California, Los Angeles, CA, United States

**Keywords:** biofeedback, telerehabilitation, electromyography, stroke, human-computer interface

## Abstract

Stroke is a leading cause of adult disability in the United States. High doses of repeated task-specific practice have shown promising results in restoring upper limb function in chronic stroke. However, it is currently challenging to provide such doses in clinical practice. At-home telerehabilitation supervised by a clinician is a potential solution to provide higher-dose interventions. However, telerehabilitation systems developed for repeated task-specific practice typically require a minimum level of active movement. Therefore, severely impaired people necessitate alternative therapeutic approaches. Measurement and feedback of electrical muscle activity *via* electromyography (EMG) have been previously implemented in the presence of minimal or no volitional movement to improve motor performance in people with stroke. Specifically, muscle neurofeedback training to reduce unintended co-contractions of the impaired hand may be a targeted intervention to improve motor control in severely impaired populations. Here, we present the preliminary results of a low-cost, portable EMG biofeedback system (Tele-REINVENT) for supervised and unsupervised upper limb telerehabilitation after stroke. We aimed to explore the feasibility of providing higher doses of repeated task-specific practice during at-home training. Therefore, we recruited 5 participants (age = 44–73 years) with chronic, severe impairment due to stroke (Fugl-Meyer = 19–40/66). They completed a 6-week home-based training program that reinforced activity of the wrist extensor muscles while avoiding coactivation of flexor muscles *via* computer games. We used EMG signals to quantify the contribution of two antagonistic muscles and provide biofeedback of individuated activity, defined as a ratio of extensor and flexor activity during movement attempt. Our data suggest that 30 1-h sessions over 6 weeks of at-home training with our Tele-REINVENT system is feasible and may improve individuated muscle activity as well as scores on standard clinical assessments (e.g., Fugl-Meyer Assessment, Action Research Arm Test, active wrist range of motion) for some individuals. Furthermore, tests of neuromuscular control suggest modest changes in the synchronization of electroencephalography (EEG) and EMG signals within the beta band (12–30 Hz). Finally, all participants showed high adherence to the training protocol and reported enjoying using the system. These preliminary results suggest that using low-cost technology for home-based telerehabilitation after severe chronic stroke is feasible and may be effective in improving motor control *via* feedback of individuated muscle activity.

## Introduction

Almost 800,000 people have a stroke each year in the United States (Virani et al., 2020), making stroke a leading cause of long-term adult disability. Recent research has shown that high doses of repeated task-specific practice may improve upper limb function in the chronic phase of stroke (>6 months after onset) (Winstein et al., 2016; Ballester et al., 2019; Ward et al., 2019). However, it is currently challenging to provide such doses in standard clinical practice due to time, physical, and economic constraints. At-home telerehabilitation services supervised by a clinician are a potential solution to provide higher-dose interventions.

Recent studies have shown that post-stroke telerehabilitation is feasible to induce positive changes and may also be as effective as in-person interventions (Dodakian et al., 2017; Cramer et al., 2019). However, repeated task-specific practice interventions, including those that use telerehabilitation services, typically require a minimum level of active movement (Winstein et al., 2003; Cramer et al., 2019; Ward et al., 2019). Therefore, severely impaired survivors necessitate alternative therapeutic approaches. Biofeedback of relevant physiological activity [e.g., *via* electroencephalography (EEG) or electromyography (EMG)] are promising approaches that have been implemented in the presence of minimal or no volitional movement and could be incorporated with telerehabilitation (Armagan et al., 2003; Wright et al., 2014; Soekadar et al., 2015; Remsik et al., 2016). Specifically, training to reduce unintended co-contractions of the impaired hand may be a targeted intervention to improve motor control in severely impaired populations (Donoso Brown et al., 2014; Mugler et al., 2019).

Recently, we tested the feasibility of a multimodal biofeedback system (REINVENT) that can interchangeably operate as a brain-computer interface (BCI) or a muscle-computer interface (MCI), and that integrates immersive virtual reality (VR) for upper limb chronic stroke rehabilitation (Vourvopoulos et al., 2019; Marin-Pardo et al., 2020). Both pilot studies with the REINVENT system showed promising results in terms of user satisfaction and moderate improvement in clinical assessments of stroke recovery. Ongoing limitations for in-person research (e.g., sanitary precautions due to the COVID-19 pandemic) and the growing interest in incorporating telerehabilitation services into standard clinical practice prompted us to develop a low-cost, portable version of our system (Tele-REINVENT) (Marin-Pardo et al., 2021). Additionally, using mobile devices for at-home telerehabilitation may further improve care access for underserved populations limited, for example, by their proximity to rehabilitation centers, mobility level, access to transportation services, and cost of care (Marzolini et al., 2016; Health equity in telehealth, Health equity in telehealth).

Here, we explored the feasibility of using Tele-REINVENT with five stroke survivors in the chronic stage of recovery that presented with severe hemiparesis (e.g., < 20° of voluntary wrist motion) and unintended antagonist activation during attempted wrist movements. We asked them to complete 30 remote training sessions, where our system reinforced extensor activation without simultaneous activation of flexor muscles. To quantify changes before and after training, we used a battery of standard clinical assessments in combination with a test of muscle control to evaluate generalized functional improvements. Additionally, we evaluated changes over time during the training intervention by quantifying the activation of agonist and antagonist muscles and their overall contribution toward less unintended coactivation. Finally, we included measurements of corticomuscular coherence (CMC) to investigate possible changes in brain organization before and after training. Although variable across stroke survivors (e.g., in terms of magnitude and localization), previous research has suggested that CMC could be interpreted as a proxy of tract integrity and neural recovery (Rossiter et al., 2013; Krauth et al., 2019; Liu et al., 2019; Marin-Pardo et al., 2020).

Overall, the purpose of this work is to explore the feasibility of Tele-REINVENT to safely provide higher doses of repeated task-specific practice during at-home upper limb training. Previous literature has identified that adherence to post-stroke home programs ranges from 50 to 100% (Jurkiewicz et al., 2011; Donoso Brown et al., 2014; Dodakian et al., 2017; Cramer et al., 2019). We designed Tele-REINVENT to overcome some of the known limitations of technology-based at-home training programs [e.g., portability and ease of use—further detailed in Marin-Pardo et al. (2021)]. Therefore, we hypothesized that at-home reinforcement of EMG activity using Tele-REINVENT in participants with severely impaired motor function of the upper extremity would be safe, feasible, and enjoyable for participants to use with high adherence (e.g., at least 80%). Moreover, as previous research by us and others has shown, not all participants will respond to training interventions (Kwakkel et al., 2016; Vourvopoulos et al., 2019; Ward et al., 2019; Marin-Pardo et al., 2020). Therefore, we hypothesized that training with Tele-REINVENT would produce improvements in clinical assessments for some but not all participants. Finally, we expect to show evidence of improved neuromuscular control, measurable as increased muscle recruitment individuation (i.e., group-specific activation with reduced unintended coactivation) and improved CMC.

## Materials and methods

### Participants

For this case series, we recruited five adult stroke survivors in the chronic phase of recovery (>6 months since onset). Inclusion criteria required that participants presented with moderate to severe upper extremity hemiparesis and residual hand function (e.g., < 20 degrees of active wrist or finger extension and enough muscle activity to measure with electromyography). Furthermore, participants were not taking anti-spasticity medication and had no significant vision loss (corrected vision was acceptable), receptive aphasia, hand contractures, or a secondary neurological disease. Finally, none of the participants were receiving additional physical or occupational therapy targeting wrist movements; however, regular exercise (e.g., training at the gym) was allowed. Participants completed a screening session prior to enrollment to ensure their compliance with these criteria. Additionally, we screened for cognitive impairment, as severe impairment might impede participation in the study tasks. The experimental protocol was approved by the Institutional Review Board of the University of Southern California (reference number: HS-17-00916, approved on 7/20/2021) and all participants provided written informed consent in accordance with the Declaration of Helsinki. A summary of the participant demographics is presented in Table 1.

**Table 1:** Participant demographics and baseline evaluations.

**Participant**	**Age**	**Onset (months)**	**Paresis**	**FMA**	**ROM-WE (°)**	**MRS**	**MOCA**
1	61	156	Left	20	10	2	22
2	73	130	Left	19	10	2	22
3	58	14	Left	23	5	2	20
4	57	25	Left	40	22	1	21
5	44	61	Right	25	−15	2	21

FMA, Fugl–Meyer Assessment of the upper extremity; ROM-WE, Range of Motion during active Wrist Extension; MRS, Modified Rankin Scale; MOCA, Montreal Cognitive Assessment.

### Study timeline

**Figure 1:**
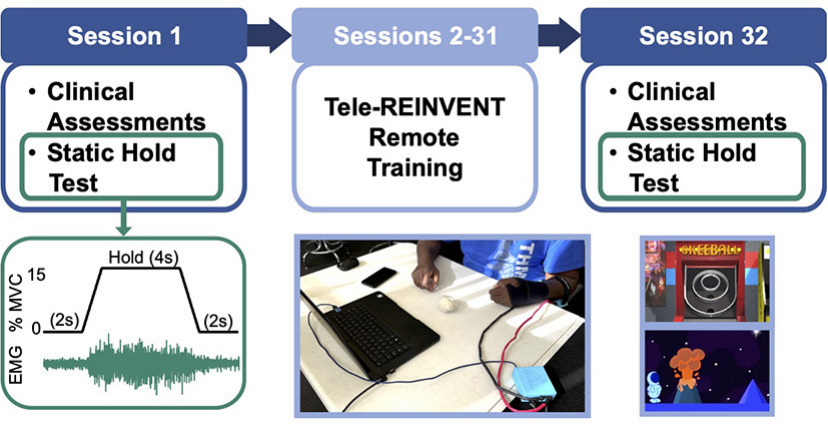
Experimental protocol. **(Top)** Timeline of the 32-sessions program. Participants were asked to complete 30 remote sessions (sessions 2–31) targeting training wrist extension activation of the more affected arm. We included pre-, and post-intervention in-person assessments in sessions 1 and 32, respectively. These comprised a battery of standard clinical assessments and a test of neuromuscular control (i.e., repeated static holds with simultaneous electromyography (EMG) and electroencephalography (EEG) recordings). **(Bottom, left)** Muscle control test (static hold). We tested control over wrist extensor and flexor muscles with a task where participants were asked to maintain a constant level of 15% of a maximal voluntary contraction (MVC) for twelve 4-second epochs while receiving feedback of their muscle EMG amplitude on a computer screen. This test was used to quantify task performance and coherence between brain and muscle signals. **(Bottom, middle)** Tele-REINVENT system (in a typical session), consisting of a laptop computer, a pair of active bipolar EMG sensors, and an acquisition box to digitize the EMG signals. **(Bottom, right)** Screenshots of two Tele-REINVENT training games used. These games were developed to actively encourage wrist extension movements by providing feedback of adequate patterns of muscle activation (i.e., avoiding unintended coactivation of antagonistic muscles). Successful trials (i.e., when EMG from the extensor muscle was greater than the flexor muscle) were reinforced *via* different game mechanics (e.g., character movement or points awarded).

We tested the functional outcomes and feasibility of using the Tele-REINVENT system with stroke survivors during at-home training sessions. An outline of the study timeline is shown in Figure 1, and each component is detailed below. Potential participants were screened for cognitive impairments using the Montreal Cognitive Assessment MOCA (Nasreddine et al., 2005) and with a test of EMG amplitude during an in-person visit before enrolling them in the study. Those who did not present a significant cognitive impairment likely to impair their ability to use Tele-REINVENT (i.e., having a MOCA score below 20 points) and that could maintain a minimum level of extensor EMG activity (i.e., hold 30% of a prerecorded maximum for ten 4-second trials) were enrolled in the remote training protocol, where each participant was asked to complete thirty 1-h remote training sessions over 6 weeks. These sessions were performed independently or remotely monitored by the research team (e.g., by an occupational therapist or a research engineer), as described below. We evaluated participants' improvements with physiological and clinical assessments of upper limb function during pre- and post-training in-person visits to our laboratory (sessions 1 and 32). Furthermore, we evaluated their muscle control with a static hold test. In this test, participants used feedback of their wrist extension and flexion EMG amplitude to follow a target level of activation (Figure 1). During this tracking test, we simultaneously recorded EEG over the ipsilesional and contralesional motor cortices to evaluate their CMC. We made every effort to perform in-person evaluations within < 3 h per session (breaks included) to avoid unnecessary burden on our participants. In sessions 2–31 participants were asked to complete an hour of remotely monitored or independent at-home training sessions of individuated wrist extension, as detailed below. We included an additional in-person visit after completion of half of the remote sessions to ensure proper location of the EMG sensors and no adverse effects for the remainder of the experiment. As mentioned above, since we were interested in functional changes before and after 30 sessions of remote training, we excluded this additional visit from our present analyses.

### At-home training (sessions 2–31)

Our training paradigm builds on the protocol and telerehabilitation system that we described in our previous work (Marin-Pardo et al., 2020, 2021). Briefly, our Tele-REINVENT system consists of a laptop computer with all necessary programs preloaded, configured, and displayed in an easy-to-use manner, a pair of low-cost EMG sensors (Figure 1), and a package of disposable electrodes and alcohol wipes. To synchronize, record, and transfer data between the different modules, we used the Labstreaming Layer (LSL) protocol and recorder (Swartz Center for Computational Neuroscience, Swartz Center for Computational Neuroscience). EMG signals were processed and analyzed in real time with custom scripts in Matlab (R2021a, The Mathworks, Natick, USA) to quantify an activation ratio of one extensor and one flexor muscle. Both the user interface and the games were developed using the Unity game engine (v2020.1.11, Unity Technologies, San Francisco, USA) and rendered on the laptop screen. These games were developed aiming to reward extensor activation when it was produced without flexor coactivation. Further details regarding game mechanics and system modules can be found in our previous work (Marin-Pardo et al., 2021). Below, we present a summary of the games implemented in this study (Figure 1).

- **SkeeBall**. In this game, we use the activity from wrist muscles to move a ball to different targets according to the ratio of activity between the extensors and flexors. Calculated ratios are mapped to score values and trigger the corresponding animation of a hand hitting the ball and the ball moving to the appropriate score ring.
- **Blinko**. Here we use the ratio of activity from the wrist extensors and flexors to move a character holding a disc across the top of a vertical game board. For each trial, the player attempts to move the character left and right with wrist extension and flexion before time runs out. Then, the character drops the disc and receives the points that correspond with the slot where the disc landed.
- **Planet Jump**. This is a side-scrolling game where a character moves along two dimensions to avoid obstacles within a finite course. For time periods where muscle activity is below an activation threshold (i.e., at rest), the character runs left to right across the environment. Otherwise, the ratio of muscle activity is translated as jumping commands for extension and stopping commands for flexion.

We used the proposed system to train individuated muscle control with the following protocol. First, participants completed a detailed orientation in session 1, including a step-by-step demonstration on how to place the EMG sensors and use the system. Additionally, we used a surgical marker pen to mark adequate sensor locations. Then, the first week of remote sessions (2–5) were monitored by the research team (e.g., an occupational therapist or a research engineer) to ensure the system worked properly and that participants were confident using it on their own. Subsequent sessions (6–31) were mostly independent, with the research team monitoring two sessions of each week. This allows us to ensure the system worked properly, make necessary adjustments to the game configurations (e.g., number of trials per game or activation threshold), and interact with the participants. This was particularly important, as research shows that when using home-based technology for rehabilitation, participant engagement and adherence are highly dependent on the involvement of clinicians and researchers (Chen et al., 2019; Cramer et al., 2019; Feldner et al., 2020). Additionally, the Tele-REINVENT kit included a printed manual with detailed instructions on how to use and setup the system, a surgical marker pen to mark adequate sensor positioning, and additional objects to increase comfort and participation during the session (e.g., a low-stiffness ball, a pool noodle, and a towel). Each remote session is described below.

For enhanced simplicity and consistency, the system interface included tutorial videos to remind the participants how to start a telerehabilitation session, plug in the acquisition device, and position the sensors over the targeted muscles. Prior to the start of all training sessions, a calibration video guided the participants through wrist and hand movements, including gross grasp, wrist extension, and wrist flexion. Upon completion of the recording, a Matlab script calculated the mean maximum amplitude of each EMG signal during a 250-millisecond moving window after processing the signals with a 15–450 Hz bandpass filter and full-wave rectification. Then, the participants selected which game they would play. This launched another Matlab script that would continuously calculate ratios of extensor activity as described in **Equation 1**:


$$\begin{array}{l} ER\;=\;\frac{EMG_{extensor}}{EMG_{extensor}+EMG_{flexor}}\; \end{array}$$

where each EMG signal corresponds to averaged activity of 250 ms of each muscle after preprocessing and normalization to the calibration activity. In this equation, values closer to 1 would indicate that the extensor muscles were comparatively more active than flexor muscles. Similarly, values closer to 0 would indicate higher flexor activity and values closer to 0.5 would indicate similar recruitment from both muscles. Finally, online feedback was provided as the game interactions described above. We asked participants to play their preferred combination of games for at least 1 h of training per session for a total of thirty sessions. To quantify functional changes after training, we used the assessments described below.

### Clinical assessments (sessions 1 and 32)

Two trained occupational therapists performed standard clinical assessments during the pre- and post-training evaluations. Functional assessments were video recorded and independently evaluated by both occupational therapists to ensure adequate inter-rater reliability. The complete set of assessments used in this study included the following:

- Montreal Cognitive Assessment (MOCA). This is an assessment of mild cognitive impairment that evaluates visuospatial abilities, memory, attention, concentration, language, and orientation, and provides a score that ranges from 0 (greatest impairment) to 30 (no impairment) (Nasreddine et al., 2005). Typically, mild cognitive impairment is defined below 26 (Nasreddine et al., 2005) or, more recently, 23 points (Carson et al., 2018). However, other computer-based training paradigms have successfully recruited stroke participants with mean scores as low as 20 points (Cramer et al., 2019; Yeh et al., 2019; Ozen et al., 2021). Therefore, to broaden the pool of participants that could qualify for our study, we used a 20-point threshold as inclusion criterion.
- Fugl–Meyer Assessment of the upper extremity (FMA). This scale measures sensorimotor impairment of the upper limb following a hemiplegic stroke, including movement, coordination, and reflexes, and provides a score that ranges from 0 (greatest impairment) to 66 (no impairment) (Fugl-Meyer et al., 1975).
- Action Research Arm Test (ARAT). This assessment measures functional performance of the upper limb in terms of the ability to manipulate objects with different sizes, weights, and shapes, and provides a score that ranges from 0 (greatest impairment) to 57 (no impairment) (Lyle, 1981).
- Wrist range of motion (ROM). Using a goniometer, we recorded the maximum degrees of active wrist extension and flexion. On average, activities of daily life usually require 40–60 degrees of wrist extension and 40–60 degrees of wrist flexion (Ryu et al., 1991; van Andel et al., 2008).
- Modified Rankin Scale (MRS). This scale measures the disability of the stroke survivor based on their independence to look after themselves in daily life, providing a range from 0 (no symptoms of disability) to 5 (severe disability) (Swieten et al., 1988).
- Grip strength.

### Characterization of muscle control during EMG amplitude tracking (sessions 1 and 32)

We sought to determine whether our task-specific training protocol induced generalized functional changes in wrist muscle activity. To quantify changes in muscle control beyond task-specific performance, participants completed two tracking tasks that were distinct from training but required similar muscle activation. In the first task, participants were asked to maintain a constant level of wrist extension of their more affected hand using the EMG amplitude from an extensor muscle to follow a target of activity. As shown in Figure 1, this target corresponded to a 4-sec hold phase (plateau) of a trapezoid spanning 6 secs and was set to 15% of the tracked muscle's maximal activity (established during a prerecorded power grip). Participants completed twelve trials where EMG was rectified and smoothed with a 0.5-sec moving window to control the height of a cursor that moved left to right across the computer screen for 10 secs before looping back. The second task followed the same protocol during wrist flexion. For each tracking task, the muscle with the largest signal to noise ratio during voluntary activation was chosen to provide EMG feedback, and practice trials were provided to ensure stable performance. Importantly, this task is quasi-isometric for these participants as the required level of muscle contraction resulted in little if any overt movement of the wrist. Participants were provided with a low-stiffness ball to avoid uncomfortable curling of their fingers. We discouraged participants from actively attempting to grip the ball, but unintended gripping was allowed. Participants rested their more affected hand on a pillow for comfort for the duration of these tasks.

#### Data acquisition

We measured surface EMG signals from four muscles at 2148 Hz using a Delsys Trigno Wireless System (Delsys Incorporated, Natick, USA). EMG sensors were placed on the skin above the extensor carpi radialis longus (ECR), extensor carpi ulnaris (ECU), flexor carpi radialis (FCR), and flexor carpi ulnaris (FCU) of the more affected arm after cleaning the area with isopropyl alcohol. Proper positioning was confirmed *via* muscle palpation and signal observation during attempted wrist extension, flexion, radial and ulnar deviation, and light grip. EMG was acquired using LabRecorder, an application designed to synchronize and record data using the LSL protocol. Signals were down sampled to 1,000 Hz for offline analysis.

Additionally, we concurrently recorded EEG at 500 Hz over the left and right motor cortices using a 32-channel LiveAmp system (Brain Vision LLC, Morrisville, USA). Electrodes were positioned over a subset of the 10–10 standard placement convention (Chatrian et al., 1985) using the Brain Vision actiCAP. In this study, we only used EEG to assess corticospinal connectivity changes *via* corticomuscular coherence after EMG-based training. Thus, we only analyzed the electrodes corresponding to the frontal-central, central, and central parietal scalp locations (e.g., FC1, FC5, C3, CP1, CP5, C4, FC2, FC6, C4, CP2, and CP6), as these are on the areas associated with motor control and correspond to the locations we used in our previous studies (Vourvopoulos et al., 2019; Marin-Pardo et al., 2020). EEG signals were synchronized and acquired using LSL and LabRecorder and then interpolated to 1000 Hz for offline analysis.

#### Muscle group individuation

We quantified the level of wrist muscle individuation with ratios of activation during the EMG tracking task, i.e., the proportion of activity from the recorded extensor muscles to the total activity from all recorded muscles using **Equation 1**. This allowed us to estimate the level of muscle group recruitment during the extension and flexion tracking tasks. We used the last 3 secs of each hold phase to calculate ER values and evaluated changes in individuation before and after the training sessions.

#### Corticomuscular coherence

In addition to the calculations of muscle individuation and tracking error, we used the same time epochs to assess the synchronization between brain and muscle signals. This measure, known as corticomuscular coherence (CMC), is a frequency-domain correlation where a value of 0 indicates no correlation between signals and 1 indicates perfect correlation at a given frequency. Specifically, we were interested in evaluating changes within the beta band (e.g., 12–30 Hz), as this has been used to probe corticospinal communication, is frequently detected during static muscle contractions, and has been suggested to improve during recovery (Mima et al., 2001; Rossiter et al., 2013; von Carlowitz-Ghori et al., 2014; Krauth et al., 2019; Liu et al., 2019; Marin-Pardo et al., 2020).

We calculated CMC in the same way as in our previous work (Marin-Pardo et al., 2020). Briefly, EEG signals were first bandpass filtered between 5 and 100 Hz using a sixth order, zero-phase Butterworth filter, and re-referenced to the common average after removing noisy or bad channels identified using an artifact detection method within Matlab's EEGLAB toolbox (Delorme and Makeig, 2004; Kothe and Jung, 2016). We used channels C3 and C4 to calculate their coherence with wrist EMG signals. If one of these channels had to be excluded, a neighboring electrode corresponding to the respective sensorimotor hemisphere was used instead, as previous studies have shown that CMC is not precisely localized during unimanual actions in this study population (Rossiter et al., 2013; Krauth et al., 2019). EMG signals were first bandpass filtered between 15 and 450 Hz, then we used the Hilbert transform to obtain their amplitude envelope (Boonstra and Breakspear, 2012; Farina et al., 2013). Finally, we normalized the resulting signals to have zero-mean and unit variance (Baker, 2000; Halliday and Rosenberg, 2000). For each task, pooled CMC (Amjad et al., 1997) was calculated between the pair of muscles involved in the task (i.e., wrist flexors or wrist extensors) and the ipsilesional and contralesional hemispheres. All trial epochs for a single subject were first concatenated, and then coherence was calculated using the mscohere function in Matlab, using 512 ms Hann-windowed segments with 75% overlap. A 95% confidence level for each coherence profile was calculated using **Equation 2** (Rosenberg et al., 1989):


$$\begin{array}{l} CL\;=\;1-0.05^{\frac{1}{L-1}}\; \end{array}$$

where L is the number of segments used to calculate coherence, adjusted for tapering and overlap (Kattla and Lowery, 2010). For a group-level analysis, we repeated the above procedure, concatenating data from all five participants. This allowed us to use the same statistical methods to evaluate group-level data as for individual coherence profiles (Amjad et al., 1997).

### Statistical analyses

We utilized custom scripts in Matlab (R2021a, The Mathworks, Natick, USA) and R (R Foundation for Statistical Computing, Vienna, Austria) for offline signal processing and statistical analyses. We anticipate that some people will improve across different metrics. However, we do not expect that these changes will be consistent for the group, as previous literature from ourselves and others have consistently shown that participants demonstrate variable improvements after similar training interventions (Ramos-Murguialday et al., 2013; Mugler et al., 2019; Vourvopoulos et al., 2019; Marin-Pardo et al., 2020). Therefore, we focus our analyses on individual changes and report results at the group level for completeness.

#### Behavioral changes in clinical assessments

Because the distribution of the data is not normally distributed, we used paired Wilcoxon signed-rank tests to identify consistent changes across the group in FMA, ARAT, ROM, and grip strength. As these assessments quantify impairments in different domains, we considered these group-level tests as independent and thus significant at the p < 0.05 level without correction. Additionally, we used Pearson's correlations to assess possible correlations between functional improvements and time after stroke onset, setting a significance threshold at p < 0.05.

#### Muscle control changes during EMG amplitude tracking

Using **Equation 1**, we calculated ratios of muscle-group activity to quantify the involvement of both muscle groups while performing the extension and flexion tracking tasks. We calculated mean ER values for each trial and evaluated differences between pre- vs. post-training assessments with signed-rank tests. Similarly, group-level effects were evaluated using paired signed-rank tests of participants' mean values for each task. The significance level for individual and group-level differences was set at p < 0.05.

#### Neuromuscular changes following training

Similar to our previous work (Marin-Pardo et al., 2020), we evaluated changes in CMC at the group level, with a Z-score difference of coherence (Rosenberg et al., 1989; Laine et al., 2014) (pre vs. post) at each frequency for each task (flexion and extension) and hemisphere (ipsilesional and contralesional) using the formula in **Equation 3**:


$$\begin{array}{l} Z_{diff}\;=\;\frac{Fz_{post}-Fz_{pre}}{\sqrt{\frac{1}{2L_{post}}+\frac{1}{2L_{pre}}}}\; \end{array}$$

where FZ is the Fisher-transformed coherence value [i.e., atanh(sqrt(coherence)] and L represents the degrees of freedom, calculated as described for **Equation 2**. This provides a standard Z score for the difference in coherence between sessions 1 and 32, for every frequency. Then, we created a composite Z-score for the beta band using Stouffer's Z-score method (Kilner et al., 1999). Composite Z-scores with an absolute value above 1.96 are considered significant at the 5% confidence level.

#### EMG activity across training sessions

To assess flexor and extensor amplitude changes over sessions, we took normalized EMG signals and calculated the averaged amplitude for each muscle during game play. First, signals were inspected manually (*via* visual inspection) and automatically (*via* examination of signal-to-noise ratios) to remove files with poor signal quality. Records with acceptable signals were filtered, rectified, and normalized as described for online processing, and trials containing activity periods of at least 1 sec for either muscle were concatenated. Then, we calculated each muscle's mean normalized amplitude for each session and used these values to calculate the respective ER. Finally, we used Pearson correlations to evaluate changes in activity over time. We considered correlations with p < 0.05 as significant for individual and group tests.

## Results

We evaluated feasibility, safety, and acceptability, as well as functional changes during pre- vs. post-training to characterize improvements induced by a 30-session training protocol.

### Feasibility, safety, and acceptability

All participants completed more than 85% of the remote sessions (average: 90.7% ± 5.5%), without adverse effects, including pain or general discomfort. All participants reported enjoying using the system, that it was easy to use and that, having the chance, would like to continue using it. On average, setup and calibration took < 10 mins once the participants were familiar with the system (e.g., after three sessions). Furthermore, all participants considered that having two monitored sessions per week was sufficient (e.g., for social encouragement and technical support), allowing them to successfully complete the independent sessions. Overall, all participants reported having a positive experience. Some participants qualitatively noted modest additional benefits in their arm function (e.g., more and faster movements), as well as reduced muscle tone, increased hand sensation, increased attention to and use of the affected arm, and better sleep quality. These qualitative observations were based on our interactions with the participants and analysis of post-study semi-structured interview data. However, details of these qualitative findings are beyond the scope of this manuscript and will be further discussed in future work.

### Behavioral changes in clinical assessments

**Figure 2:**
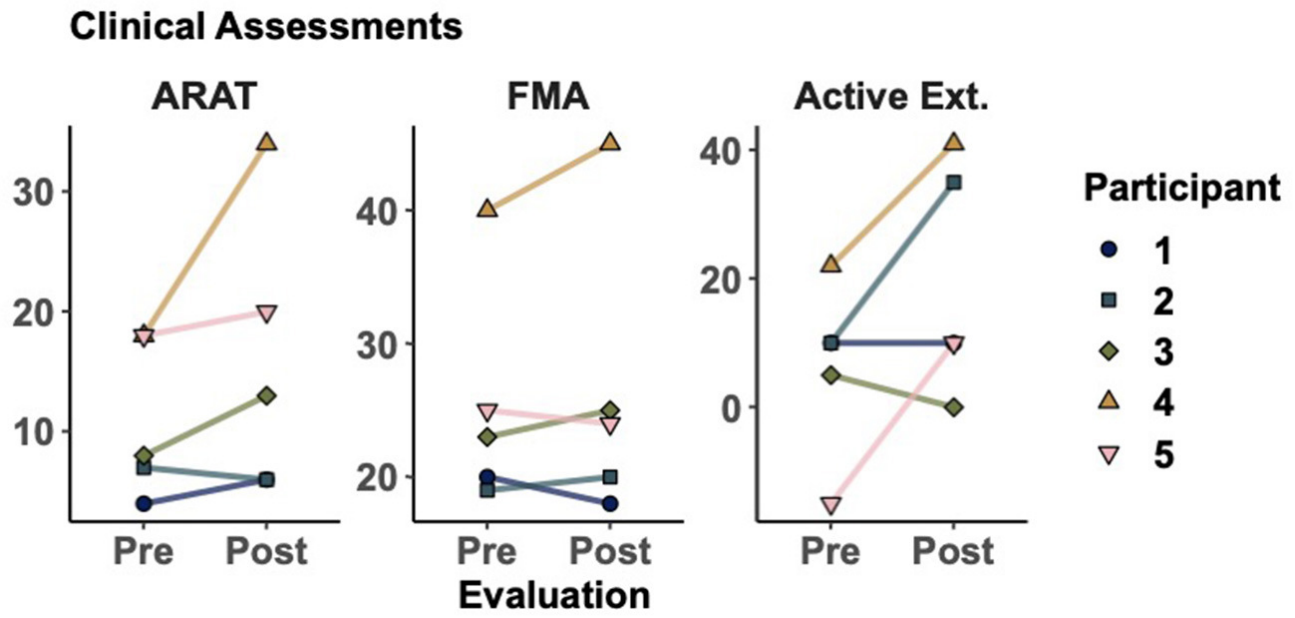
Clinical assessments before and after training. Markers represent the score for each participant as evaluated in sessions 1 and 32. Action research arm test (ARAT), Fugl-Meyer assessment of the upper extremity (FMA), active range of motion during wrist extension. Additional results can be seen in Table 2.

As seen in Table 2, all participants showed improvements in at least one of the five clinical assessments measured. Figure 2 shows the most representative changes. At the individual level, three participants showed improvement in their active extension (Participants 2, 4, and 5) and active flexion (Participants 3, 4, and 5). Furthermore, three participants showed improvements in the FMA and four in the ARAT. Notably, Participant 4 improved beyond the minimum clinically important difference (MCID) in ARAT [difference of 16 points, MCID>5.7 (van der Lee et al., 2001)] and FMA [difference of 5 points, MCID>4.25 (Page et al., 2012)]. However, no changes were statistically significant at the group level. Finally, we evaluated whether participants with less time since stroke onset showed greater improvements (i.e., greater change between pre- and post-intervention measurements). However, we did not find significant correlations between time since onset and clinical measurements, which may be due to the small sample size.

**Table 2:** Clinical assessment scores of 5 chronic stroke survivors before and after training with Tele-REINVENT.

	**ARAT**		**FMA**		**ROM AE**		**ROM AF**		**Grip (kg)**	
**Participant**	**Pre**	**Post**	**Pre**	**Post**	**Pre**	**Post**	**Pre**	**Post**	**Pre**	**Post**
1	4	6	20	18	10	10	15	15	5.8	4.7
2	7	6	19	20	10	35	30	25	5.4	8.9
3	8	13	23	25	5	0	35	45	2.5	8.3
4	18	34	40	45	22	41	35	43	7.7	7.5
5	18	20	25	24	−15	10	0	10	7.3	5.3

ARAT, Action research arm test; FMA, Fugl-Meyer Assessment of the upper extremity; ROM, Ranges of Motion, AE, Active Extension, AF, Active Flexion.

### Muscle control changes during EMG amplitude tracking

**Figure 3:**
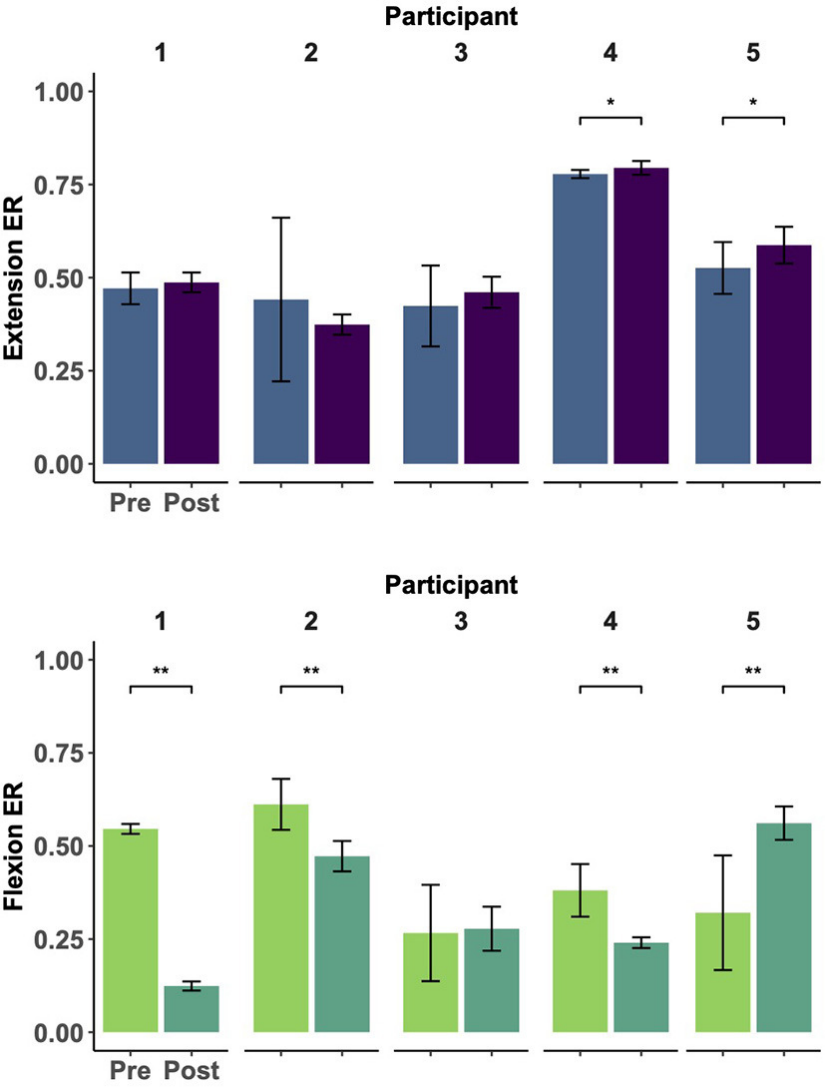
Muscle group individuation during EMG amplitude control (ramp-and-hold tracking tasks). Each panel represents the individual changes (Participants 1–5, left to right) of muscle group individuation [quantified as the ratio of extensor to total activity (ER)] at constant levels of extension **(top)** and flexion **(bottom)** tracking before and after 30 EMG training sessions. Notably, remote EMG training sessions did not require a constant level of EMG activation and did not provide explicit error-based feedback. ER values closer to 1 indicate higher extensor activity whereas values closer to 0 indicate higher flexor activity. Improvements were seen in individuated recruitment for both tracking tasks (e.g., higher individuation and lower variability). * indicates a significance of *p* < 0.05, ** indicates significance of p < 0.001. Statistical results can be seen in Table 3.

As seen in Figure 3, participants showed trends of improved motor control, measured by greater muscle individuation for the extension and flexion tracking tasks (i.e., ER values closer to 1 for the extension task and closer to 0 for the flexion task). At the individual level, two participants had a significant improvement in the extension task (*p* = 0.004 for Participant 4, and *p* = 0.033 for participant 5). Similarly, four participants showed significant improvements in the flexion task (*p* < 0.01 for Participants 1, 2, 3, and 4). However, this change was not significant at the group level for either task. Notably, all participants showed reduced variability (i.e., decreased standard deviations) in both tracking tasks. Results of individual tests are shown in Table 3.

**Table 3:** Muscle individuation during extension and flexion tracking.

	**Extension**			**Flexion**		
**Participant**	**p**	**ER pre**	**ER post**	**p**	**ER pre**	**ER post**
1	0.243	0.47 ± 0.04	0.49 ± 0.03	< 0.001^*^	**0.55** **±0.01**	**0.12** **±0.01**
2	0.291	0.44 ± 0.22	0.37 ± 0.03	< 0.001^*^	**0.61** **±0.07**	**0.47** **±0.04**
3	0.946	0.42 ± 0.11	0.46 ± 0.04	0.479	0.27 ± 0.13	0.28 ± 0.06
4	0.004^*^	**0.77** **±0.01**	**0.79** **±0.02**	< 0.001^*^	**0.38** **±0.07**	**0.24** **±0.01**
5	0.033^*^	**0.53** **±0.07**	**0.59** **±0.05**	< 0.001^*^	**0.32** **±0.15**	**0.56** **±0.05**

Signed-rank tests for muscle group individuation of five chronic stroke survivors during an EMG extension tracking task before and after training with Tele-REINVENT. Mean individuation (ER) ± standard deviations included for pre- and post-intervention evaluations.

* and bold fonts indicate significance of p < 0.05. ER values closer to 1 indicate higher activation of extensor muscles while values closer to 0 indicate higher activation of flexors. Values closer to 0.5 indicate a similar level of activation for both muscle groups.

### Changes in corticomuscular coherence following training

**Figure 4:**
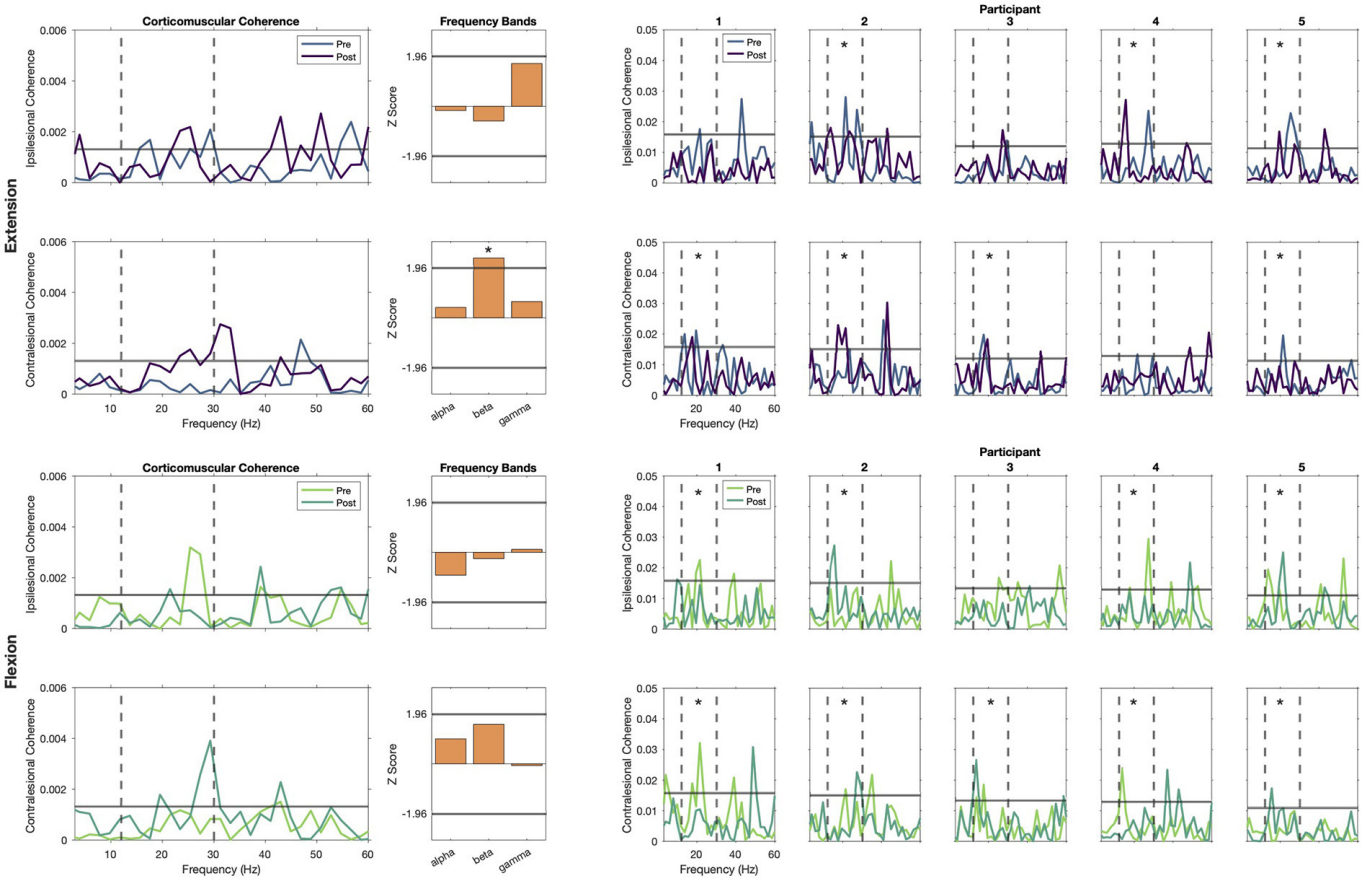
Corticomuscular coherence (CMC) during static extension and flexion. **Left** show the group-level pooled coherence for extension **(top)** and flexion **(bottom)** tasks, with activity pre-REINVENT in lighter colors and post-REINVENT in darker colors. Note that significant coherence is present during extension **(top)** before and after training in the beta band at the ipsilesional hemisphere, and that there is a significant increase in the contralesional hemisphere. Similarly, beta band coherence during flexion **(bottom)** seems to shift from the ipsilesional toward the contralesional hemisphere. **Middle** show bar plots of pooled-coherence spectra and represent the composite group difference in coherence before vs. after training within alpha (8–12 Hz), beta (12–30 Hz), and gamma (30–50 Hz) frequency bands. Asterisks denote significant changes in a frequency band before vs. after training. **Right** show individual profiles (Participants 1–5, **left** to **right**) of coherence for extension **(top)** and flexion **(bottom)** tasks. Asterisks in individual plots note participants that showed significant CMC in the beta band before or after training. Top rows of each panel show plots for ipsilesional electrodes and bottom rows show contralesional electrodes. Coherence spectra within 0 and 60 Hz are shown in all plots, including vertical dashed lines indicating boundaries of the beta band and a solid horizontal line to indicate significant coherence.

Individual coherence profiles showed high variability across participants, frequencies, and conditions. As seen in the **top right panel** of Figure 4, Participants 2, 4 and 5 showed significant coherence peaks in the ipsilesional hemisphere before and after training. Similarly, significant peaks after training are observed for Participants 1, 2, and 3 in the contralesional hemisphere. Our analysis of pooled coherence across participants suggests that during wrist extension, the only frequency band that had a significant increase of coherence after training was the beta band of the contralesional cortex (Figure 4, **top left panel**, Z-score pre-post difference = 2.35, *p* = 0.018). Importantly, this does not imply lack of activity from the ipsilesional cortex, as the pooled coherence profiles show significant coherence before and after training. For completeness, we also analyzed changes in the alpha and gamma frequency bands, where a non-significant trend of increased coherence in the gamma band is seen for the ipsilesional cortex (Z-score difference = 1.68, *p* = 0.093). As seen in the **bottom right panel** of Figure 4, we also identified peaks of coherence after training in the beta band for the ipsilesional (Participants 2 and 5) and contralesional hemispheres (Participants 2, 3, and 5) during the flexion task, but these changes were not consistent according to the pooled coherence analysis. We detected a non-significant trend in the beta band in the contralesional hemisphere (Figure 4, **bottom left panel**, Z-score pre-post difference = 1.55, *p* = 0.121).

### Increased EMG individuation across sessions

**Figure 5:**
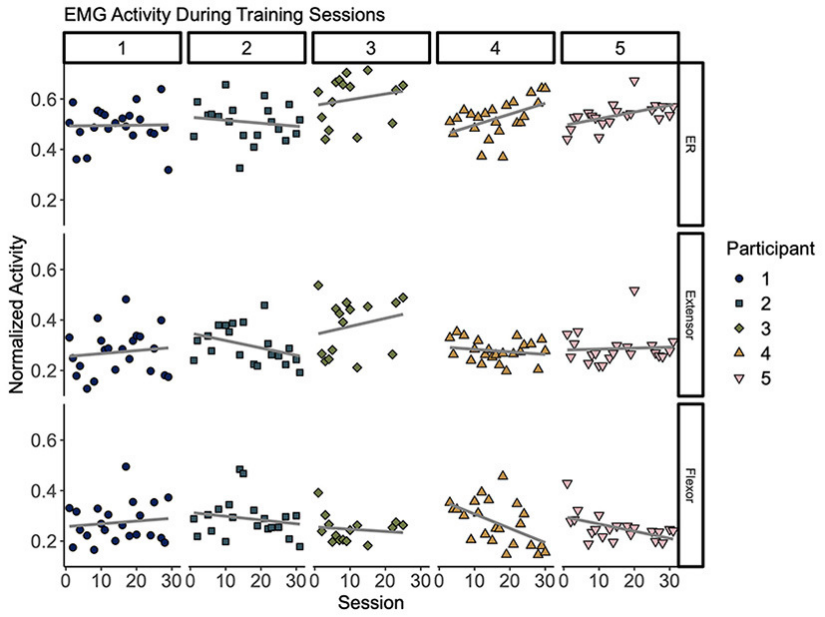
Muscle activity during remote training sessions. Each column (1–5) represents per-session averages (markers) of muscle activity (rows) during training for each participant. **(Top)** Muscle individuation (ER) where values closer to 1 indicate higher extension activity and values closer to 0 indicate higher flexion activity. **(Middle)** Normalized extensor electromyography (EMG) activity. **(Bottom)** Normalized flexor EMG. Overall, these plots suggest modest trends of improved individuation over time (**top row**). Changes of ER over time showed a significant increase for Participants 4 (*p* = 0.018) and 5 (*p* = 0.013). This change was accompanied by a decrease of flexor activity over time (*p* = 0.009 for Participant 4 and *p* = 0.016 for Participant 5). Best fit lines are included to visualize trends across sessions in gray.

As shown in Figure 5, measures of muscle activity suggest higher individuation over time (top row). Overall, muscle individuation appears to increase during training for Participants 3, 4, and 5. These changes were significant for Participants 4 (*p* = 0.018) and 5 (*p* = 0.013). These changes were accompanied by a significant decrease of flexor activity for Participants 4 (*p* = 0.009) and 5 (0.016). At the group level, we observed a significant decrease of overall flexor activity (*p* = 0.045). Table 4 shows correlation results for all five participants.

**Table 4:** Changes of muscle activity over time during remote training sessions.

	**ER**		**Extensor**		**Flexor**	
**Participant**	**rho**	**p**	**rho**	**p**	**rho**	**p**
1	0.02	0.937	0.12	0.607	0.12	0.587
2	−0.15	0.536	−0.38	0.100	−0.18	0.448
3	0.19	0.502	0.23	0.417	−0.13	0.642
4	**0.49**	0.018^*^	−0.19	0.389	–**0.53**	0.009^*^
5	**0.53**	0.013^*^	0.06	0.807	–**0.52**	0.016^*^

Pearson correlations of five chronic stroke survivors during EMG biofeedback training with Tele-REINVENT. Bold fonts and

* indicate significance of p < 0.05.

## Discussion

We explored the use of an EMG-based telerehabilitation program that attempts to improve severe motor deficits after chronic stroke *via* training of muscle activation without unintended activation of antagonistic muscles. We found that 30 1-hr sessions of combined supervised and unsupervised remote training can induce positive outcomes in motor function in a pilot study with five stroke survivors. Although variable across participants, similar to our previous studies (Vourvopoulos et al., 2019; Marin-Pardo et al., 2020), these results suggest that our telerehabilitation system can elicit functional changes in severely impaired individuals. These changes were measurable with standard clinical assessments, the proportion of activation of agonistic and antagonistic muscles, and modest improvements in corticomuscular connectivity. Additionally, all participants reported an overall positive experience and showed high adherence to the proposed training protocol.

### Clinical assessments

We hypothesized that we would observe improvements in clinical assessments after training. Much effort has been invested in developing novel and effective non-invasive rehabilitation treatments. However, previous research has shown that, even for treatments with proven efficacy and narrow inclusion criteria, not all participants will positively respond to interventions (Kwakkel et al., 2016; Ward et al., 2019). Accordingly, more investigation is needed to better understand which elements (e.g., population characteristics and neural mechanisms engaged in repeated practice) could better predict which participants might benefit from targeted rehabilitation approaches (Winstein et al., 2016; Bernhardt et al., 2017). Similarly, we expected that some, but not all, of our participants would show improvements in clinical assessments. Subsequently, due to our limited sample size, we expected that inconsistent changes (i.e., not seen across all participants) would result in non-significant changes at the group level. Furthermore, previous research has suggested that individuals presenting with less time after stroke onset might show greater improvement after training interventions (Ballester et al., 2019). While that could also be the case for our proposed training, we did not make a specific hypothesis about it due to the small sample of this feasibility study.

In previous work, we showed that few sessions (*n* = 7) of wrist training to encourage extension movements without unintended flexor recruitment produced moderate improvements in several clinical assessments (Marin-Pardo et al., 2020). Our results from that experiment and this new iteration had similar trends, i.e., improvement across different assessments and some participants showing changes beyond established MCIDs. Qualitatively, our current results suggest that higher training dosage might have induced greater functional changes. This can be observed in greater differences between pre- and post-training measurements, compared across participants that initially had similar levels of impairment. On average, ARAT and wrist extension changes were greater for those who participated in 30 remote training sessions, compared to those who participated in 7 laboratory-based sessions. However, we did not find a statistically significant difference between the two groups (i.e., a significant correlation between level of improvement and completed training sessions). As we lack the statistical power to explore such relationships, further research with larger samples is necessary to better quantify the effect that training dosage might have induced on functional outcomes.

Literature suggests that relatively high dosages of repeated task-specific practice can induce positive outcomes when using EMG-based technologies after chronic stroke (Mugler et al., 2019; Jian et al., 2021). In line with our results, Mugler et al. (2019) and Jian et al. (2021) showed that training to reduce abnormal muscle coactivation with similar dosage and intensity could reduce impairment after chronic stroke with comparable results to ours. Importantly, this level of exercise is already higher than what patients receive during standard clinical practice, as sessions are often limited by the time a clinician can spend on a specific limb or joint (Lang et al., 2009). However, further research with larger samples is required to allow stratification of different levels of dosage and intensity to evaluate the effect that these parameters have on the level of improvement that EMG biofeedback can induce.

It is important to note that although our training paradigm sought to specifically train wrist movements, we saw functional changes beyond the activation of the muscles we used for training. As expected, most participants improved their active ranges of motion during wrist extension and flexion. However, changes in FMA and ARAT scores are not fully explained only by improved recruitment of hand muscles, as some participants showed improvement on assessment items that required coordination of the whole upper limb. Results from our tracking task suggest that wrist extension training also allowed for more individuated control during the flexion task (see below). Therefore, we theorize that strategies our participants learned to produce isolated movement of the wrist could have also been applied to improve control of other muscle pairs, improving their overall upper limb control. However, further research is required to evaluate whether learning to better control a specific muscle group correlates with improved control of untrained muscles.

Finally, it is widely accepted that most spontaneous biological recovery plateaus within the first 6 months after stroke onset and that improvement in motor outcomes beyond this time window might be mostly driven by learning-dependent processes or compensation strategies (Kwakkel et al., 2004; Bernhardt et al., 2017). Therefore, although we did not specifically quantify spontaneous improvement with multiple baseline measurements, it is reasonable to assume that changes in motor function could be attributed to our proposed training. Future work should include multiple baseline measurements to confirm that, at enrollment, participants' recovery has indeed plateaued. Furthermore, the time after stroke for when no additional recovery can be expected is currently unknown. As noted above, increasing evidence shows that it is possible to induce positive motor outcomes even years after the cerebrovascular accident. However, it is likely that the efficacy of such approaches is correlated with the time after onset, allowing for greater improvements for people presenting in the more acute and subacute phases (Ballester et al., 2019). Similarly, it is possible that such populations might have benefitted more from our proposed program, and it would be valuable to explore this in future work. We did not, however, have a specific hypothesis regarding possible correlations between stroke onset and functional improvement in this study, as our sample limits such analysis.

### Improvements in muscle group individuation

Our next hypothesis was that our training program would improve neuromuscular control, quantified as increased muscle recruitment individuation. Accordingly, we showed that most participants improved their muscle individuation (measured as the extension ratio, ER) during in-person evaluations and remote training. First, most participants showed trends of increased individuation during the tracking task. This was seen not only as increased ER values during the extension tracking and decreased ER values during flexion tracking but also as overall decreased ER variability during both tracking tasks. Importantly, our training paradigm was designed to encourage extension movements *via* reinforcement of extension-like attempts that did not present significant flexion activation. That is, ER values that corresponded to those expected during attempted individuated extension (closer to 1) showed positive feedback that corresponded with the game goal (e.g., correct control commands or higher scores). Motor control literature refers to this as reinforcement learning, i.e., learning to produce successful motor commands by attempting to increase positive rewards (Krakauer, 2006; Maier et al., 2019). However, during the EMG amplitude tracking assessments, we provided an instantaneous difference between expected and produced values of EMG. This required a different control strategy that used error cues to correct behavior [i.e., error-based learning (Krakauer, 2006; Maier et al., 2019)]. Furthermore, although we did not provide feedback of the concurrent activation of the antagonist muscles during the tracking task, participants showed increased individuation in post-training sessions for both extension and flexion tasks. Overall, this suggests that we may have improved generalized behavior as the training and tracking tasks required similar activation patterns but were distinct in nature. However, further research is required to disambiguate changes in error-based and reinforcement-based strategies.

Additionally, some participants showed increased individuation over time during remote training sessions and this change was statistically significant for two participants. Importantly, this change was accompanied by a significant decrease of flexor activity over time and not increased activity of extensor muscles. Ellis and others (Ellis et al., 2017) showed that the flexion synergy, commonly seen in impaired stroke populations, is detrimental for reaching movements and that specifically targeting this impairment might be beneficial for arm function. Similarly, our results suggest that reducing activity of antagonist muscles (e.g., flexor muscles during our extension training protocol) may have a higher influence in increased muscle group individuation than increasing activity of agonist muscles. However, further research is needed to confirm this trend.

### Changes in corticomuscular coherence

In addition to seeing improvements in clinical assessments and muscle individuation during movement attempts, we also hypothesized that these changes would be accompanied by neuronal reorganization. We used CMC, a frequency-domain quantification of the synchrony between EEG and EMG signals, to probe such neuroplastic changes. CMC is typically interpreted as an indication of functional connectivity between neurons in the motor cortex and the motor neurons in the spinal cord (Mima and Hallett, 1999; Boonstra, 2013; Liu et al., 2019). Importantly, previous literature has identified inter-subject variability in this measurement in impaired and neurotypical populations (Rossiter et al., 2013; Chwodhury et al., 2015). However, CMC in the beta and gamma bands (12–30 and 30–50 Hz, respectively) has been shown to increase due to spontaneous and rehabilitation-induced recovery after stroke (von Carlowitz-Ghori et al., 2014; Zheng et al., 2018; Krauth et al., 2019). Furthermore, although CMC is typically located in the primary motor area contralateral to the muscle used to record EMG in neurotypical populations (Kilner et al., 1999; Rossiter et al., 2013; Liu et al., 2019), studies with post-stroke populations have shown variable localization sources, including the motor and premotor areas of both the ipsilesional and contralesional hemispheres (Rossiter et al., 2013; Krauth et al., 2019). Moreover, the inherent variability of lesion location among stroke survivors might introduce additional variability in the source localization of CMC (Rossiter et al., 2013). In this work, we analyzed CMC using electrodes over our participants' ipsilesional and contralesional motor cortices. Given our small sample and because we do not have data regarding the specific lesion locations of our participants, we chose such electrodes to better compare our current and previous findings (as discussed below). However, further research with larger samples and lesion location data (e.g., including anatomical magnetic resonance imaging) would be necessary to better understand possible correlations between CMC and lesion location.

Overall, our results showed variable changes in CMC across individuals during static holds of wrist flexion and extension. We observed significant beta band coherence in the ipsilesional hemisphere before and after training during the extension task, shown in individual and group coherence plots. Furthermore, we observed a significant increase in beta band coherence with the contralesional cortex, also shown in individual and group plots. Finally, we observed what appears to be a shift in coherence from the ipsilesional to the contralesional cortex during the flexion task; however, this change was not statistically significant. In line with our previous work (Marin-Pardo et al., 2020), we found a significant group increase in the beta band in the contralesional hemisphere after training. However, this change was not accompanied with the increase in the ipsilesional hemisphere we observed in our previous work. Differences between our previous and current results could be driven by several factors (e.g., demographics or training dosage). Although we aimed to include a similar population, the participants in our previous study presented with shorter times after stroke onset and, on average, more severe impairment. This could partially explain why our current participants showed significant coherence in the ipsilesional hemisphere before training and only showed increased coherence in the contralesional hemisphere. Additionally, our previous work explored the effects of relatively few training sessions, whereas here we tripled the number of sessions, which may have induced greater plasticity in CMC of the contralesional hemisphere. However, more research is needed to better understand the observed changes. Together, our results suggest that CMC may be used to quantify changes induced by EMG biofeedback training, and such changes may be mediated by the contralesional cortex both at early and late stages of training with accompanied ipsilesional contribution at early stages. However, further research with a larger sample is necessary to investigate possible correlations between CMC changes and other factors, such as, baseline impairment, training dosages, induced recovery, and training tasks.

### Limitations and future directions

A main limitation of this feasibility case series study is the small sample size. Future work employing remote assessments (Palsbo et al., 2007; Amano et al., 2018), in addition to remote training, would likely increase our sample size as the accessibility for people with mobility limitations may be improved. Future work could also engage a larger and more diverse sample that includes broader levels of impairment to fully characterize the recovery process potentially induced by Tele-REINVENT. A larger sample would also allow disambiguating potential correlations between improved outcomes, key elements of the intervention (e.g., dosage, intensity, and learning strategy), and demographics (e.g., baseline impairment, lesion location, lesion size, time after onset, and spasticity).

## Data availability statement

The raw data supporting the conclusions of this article will be made available by the authors, without undue reservation.

## Ethics statement

The studies involving human participants were reviewed and approved by Institutional Review Board of the University of Southern California. The participants provided their written informed consent to participate in this study.

## Author contributions

OM-P participated in the development of the biofeedback system, collected and analyzed data, and drafted the manuscript. MD and KW performed clinical assessments. CP participated in the development of the biofeedback system. KW, CP, and JP recruited participants and assisted in data collection. OM-P, MD, and S-LL interpreted the data. S-LL designed and supervised the study. All authors contributed to manuscript revision, read, and approved the submitted version.

## Funding

This research was funded by American Heart Association (Grant 16IRG26960017), the U.S. Army Research Office (Grant W911NNF-14-D-0005), the National Institutes of Health (Grant K01HD091283), the Mexican National Council for Science and Technology-CONACYT (Scholarship 625785/472472), and a USC Stevens Technology Advancement Grant Award.

## Conflict of interest

S-LL, OM-P, and CP reported having a patent pending as “2022, S-LL, OM-P, and CS. Neurofeedback rehabilitation system. International Publication No. WO 2022/183009 A1.” The remaining authors declare that the research was conducted in the absence of any commercial or financial relationships that could be construed as a potential conflict of interest.

## Publisher's note

All claims expressed in this article are solely those of the authors and do not necessarily represent those of their affiliated organizations, or those of the publisher, the editors and the reviewers. Any product that may be evaluated in this article, or claim that may be made by its manufacturer, is not guaranteed or endorsed by the publisher.
